# Disk displacement, eccentric condylar position, osteoarthrosis – misnomers for variations of normality? Results and interpretations from an MRI study in two age cohorts

**DOI:** 10.1186/s12903-016-0319-4

**Published:** 2016-11-17

**Authors:** Jens C. Türp, Anna Schlenker, Johannes Schröder, Marco Essig, Marc Schmitter

**Affiliations:** 1 Department of Reconstructive Dentistry and Temporomandibular Disorders, University Center for Dental Medicine Basel, University of Basel, Basel, Switzerland; 2 Department of Prosthodontics, University of Heidelberg, Heidelberg, Germany; 3 Section of Geriatric Psychiatry, University of Heidelberg, Heidelberg, Germany; 4 Department of Radiology, University of Manitoba, Faculty of Medicine, Winnepeg, Canada; 5 Department of Prosthodontics, Dental School of the University of Würzburg, Würzburg, Germany

**Keywords:** Image interpretation, Mandibular condyle, Medicalization, Medical overuse, Osteoarthritis, Osteoarthrosis, Overdiagnosis, Temporomandibular disorders, Temporomandibular joint disc, Terminology

## Abstract

**Background:**

Clinical decision-making and prognostic statements in individuals with manifest or suspected temporomandibular disorders (TMDs) may involve assessment of (a) the position of articular disc relative to the mandibular condyle, (b) the location of the condyle relative to the temporal joint surfaces, and (c) the depth of the glenoid fossa of the temporomandibular joints (TMJs). The aim of this study was twofold: (1) Determination of the prevalence of these variables in two representative population-based birth cohorts. (2) Reinterpretation of the clinical significance of the findings.

**Methods:**

From existing magnetic resonance imaging (MRI) scans of the TMJs that had been taken in 2005 and 2006 from 72 subjects born between 1930 and 1932 and between 1950 and 1952, respectively, the condylar position at closed jaw was calculated as percentage displacement of the condyle from absolute centricity. By using the criteria introduced by Orsini et al. (Oral Surg Oral Med Oral Pathol Oral Radiol Endod 86:489-97, 1998), a textbook-like disc position at closed jaw was distinguished from an anterior location. TMJ morphology of the temporal joint surfaces was assessed at open jaw by measuring the depth of the glenoid fossa, using the method proposed by Muto et al. (J Oral Maxillofac Surg 52:1269-72, 1994).

Frequency distributions were recorded for the condylar and disc positions at closed jaw.

Student’s *t*-test with independent samples was used as test of significance to detect differences of condylar positions between the age cohorts (1930 vs. 1950) and the sexes. The significance levels were set at 5%. First, the results from the measurement of the age cohorts were compared without differentiation of sexes, i.e., age cohort 1930–1932 versus age cohort 1950–1952. Subsequently, the age cohorts were compared by sex, i.e., men in cohort 1930–1932 versus men in cohort 1950–1952, and women in cohort 1930–1932 women men in cohort 1950–1952.

**Results:**

In both cohorts, condylar position was characterized by great variability. About 50% of the condyles were located centrically, while the other half was either in an anterior or in a posterior position. In both female cohorts, a posterior position predominated, whereas a centric position prevailed among men. Around 75% of the discs were positioned textbook-like, while the remaining forth was located anteriorly. Age had no statistically significant influence on condylar or on disc position. Conversely, comparison between the age groups revealed a statistically significant decrease of the depth of the glenoid fossa in both older cohorts. This age-dependent changes may be interpreted as flattening of the temporal joint surfaces.

**Conclusions:**

We call for a re-interpretation of imaging findings because they may insinuate pathology which usually is not present. Instead, anterior or posterior positions of the mandibular condyle as well as an anterior location of the articular disc should be construed as a variation of normalcy. Likewise, flattening of articular surfaces of the TMJs may be considered as normal adaptive responses to increased loading, rather than pathological degenerative changes.

**Trial registration:**

Not applicable.

## Background

Many descriptive epidemiological studies from different countries have shown that temporomandibular disorders (TMDs) are mostly prevalent among women in the child-bearing age [1–4]. This is also reflected in the higher degree of TMD-related treatment need among females as compared to males [5, 6].

Conversely, recent findings from a large prospective cohort study (OPPERA) revealed that the incidence rate of clinically verified temporomandibular pain was only marginally greater in women than in men, while there was no difference in the annual rate of TMD pain symptom episodes among the two sexes [7]. In contrast to musculoskeletal complaints located in other parts of the body, e.g. osteoarthritis [8], the incidence and prevalence of TMD symptoms, notably pain located in the masticatory muscles and/or the temporomandibular joints (TMJs), decrease remarkably during menopause and in older age [1, 9, 10].

In individuals with manifest or suspected TMDs, clinical decision-making as well as TMD-related prognostic statements of dentists are primarily based on somatic factors, such as the presence of temporomandibular pain, while psychosocial variables do not appear to play an important role, if any [6]. Traditionally, many dentists have focused on morphological variables to suggest etiological models and/or justify initiation of therapy, including (a) the position of the articular disc relative to the mandibular condyle [11, 12], (b) the location of the mandibular condyle relative to the temporal joint surfaces (glenoid fossa and articular eminence) [13, 14], and (c) the depth of the glenoid (mandibular) fossa, including flattening of the temporal surfaces [15, 16].

In the present investigation, these three anatomical variables were reanalyzed in two population-based birth cohorts to determine the prevalence of these variables and to (re)interpret the clinical significance of the findings. The cohorts were stratified by age and biological sex in order to investigate the possible influence of these two variables.

## Methods

### Previous work

At the beginning of the 1990s, a representative longitudinal population study of adult development based on two cohorts (*N* = 1001) born between 1930 and 1932 and between 1950 and 1952, respectively, had been initiated in two German urban regions, Heidelberg-Mannheim-Ludwigshafen (Baden-Württemberg/ Rhineland-Palatinate) and Leipzig (Saxonia) [17]. In 2005 and 2006, the participants of our study had been recruited from the two Heidelberg cohorts by asking them to participate in a magnetic resonance imaging (MRI) examination of the TMJs. Seventy-two individuals consented, 33 of whom were from the birth cohort 1930–1932 and 39 from the cohort 1950–1952. Data of two subsamples – “cohort 1930–1932” [73–76 years of age], “cohort 1950–1952” [53–56 years of age]) of the Heidelberg cohort – was selected. The distribution of the 72 participants, stratified by cohort and sex, is summarized in Table 1.

**Table 1:** Distribution of the two birth cohorts

	Number	f	m	Non-accessible, jaw closed	Non-accessible, jaw open
Cohort 1930–1932	66	30	36	3	10
Cohort 1950–1952	78	36	42	2	4
Σ TMJs	144	66	78	5	14

N: number of temporomandibular joints (TMJs), separated in TMJs from females (f) and males (m). At closed jaw, 139 TMJs were accessible, while 130 TMJs were accessible at open jaw. In the Cohort 1930–1932, one female participant could not be evaluated both at open and closed jaw

Subsequently, all subjects were examined according to the *Research Diagnostic Criteria for Temporomandibular Disorders* (RDC/TMD) [18]. Three participants reported pain in one TMJ. In five individuals, a reciprocal clicking was noticed during jaw opening, closing, and protrusion, but not upon protrusive opening. No subject had a limitation of mandibular movements.

In the Division of Radiology of the German Cancer Research Center at Heidelberg, contrast agent-enhanced (Magnevist; Schering AG, Berlin, Germany) MRI scans of the TMJs in the sagittal and coronal views were made in open and closed jaw positions. For that purpose, a 1.5 Tesla tomography (Symphony; Siemens, Erlangen, Germany) with TMJ surface coils (12 cm diameter; Siemens) was used. Imaging was carried out with a T1 2D fast low-angle shot (FLASH) sequence. For every individual, 10 scans were produced. The following settings were taken: slice thickness 3 mm, distance factor 20%, flip 30°, field of view 120 mm × 120 mm, time of repetition 208 ms, time of echo 10.2 ms, base resolution 256, phase resolution 80%, band width 70 Hz/Px. The slices were oriented rectangular to the long axis of the condyle. The magnetization-prepared gradient echo technique was chosen to enhance the contrast of the various tissues by acquiring the spins in different relaxation times. This technique optimizes imaging of the cartilage and is both time- and cost-effective. For the depiction of the closed position, the subjects had been instructed to close their jaw habitually with slight tooth contacts. For the stabilization of the open jaw position, a mechanical mouth opener (Burnett BiDirectional TMJ Device, Medrad Inc. Pittsburgh, U.S.A.) was used to fix the open jaw position and, therefore, to reduce blurred images due to mandibular motion.

### Current study

The total number of MRIs available for evaluation was 139 at closed and 130 at open jaw positions. Exclusion criteria had been low imaging quality and artifacts (Table 1). For each TMJ, one slice of the sagittal views – namely the slice located in the middle of the long axis of the condyle – was selected from the open and closed positions, respectively, for the assessment of the mandibular condyle and the articular disc. For the purpose of this paper, the coronal views were not considered.

Evaluations of the MRIs and all measurements were carried out with the digitizing software DICOM (Digital Imaging and Communications in Medicine) Works (Version 1.3.5, National Electrical Manufactures Association, Virginia, USA).

#### Position of the mandibular condyle at closed jaw

**Fig. 1:**
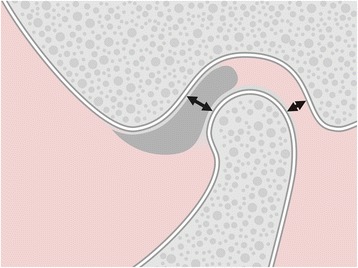
Evaluation of condylar position at closed jaw: Determination of the smallest anterior and posterior joint spaces

The condylar position at closed jaw was calculated as percentage displacement of the condyle from absolute geometric centricity. Both the smallest anterior and posterior distances between mandibular condyle and glenoid fossa (i.e., the narrowest anterior and posterior interarticular widths) were determined [19, 20] (Fig. 1). Then, the individual condylar position (CP) was calculated according to the following formula [19, 20]:

$$ \mathrm{C}\mathrm{P}=\frac{\mathrm{posterior}\hbox{-} \mathrm{anterior}}{\mathrm{posterior}+\mathrm{anterior}}\times \kern0.5em 100\% $$

The result was interpreted according to the recommendations by Ren et al. [21]:

- 0: absolute centric position of the condyle.
−12% - +12%: centric position of the condyle
- < −12%: posterior position of the condyle
- > +12%: anterior position of the condyle

#### Position of the articular disc at closed jaw

**Fig. 2:**
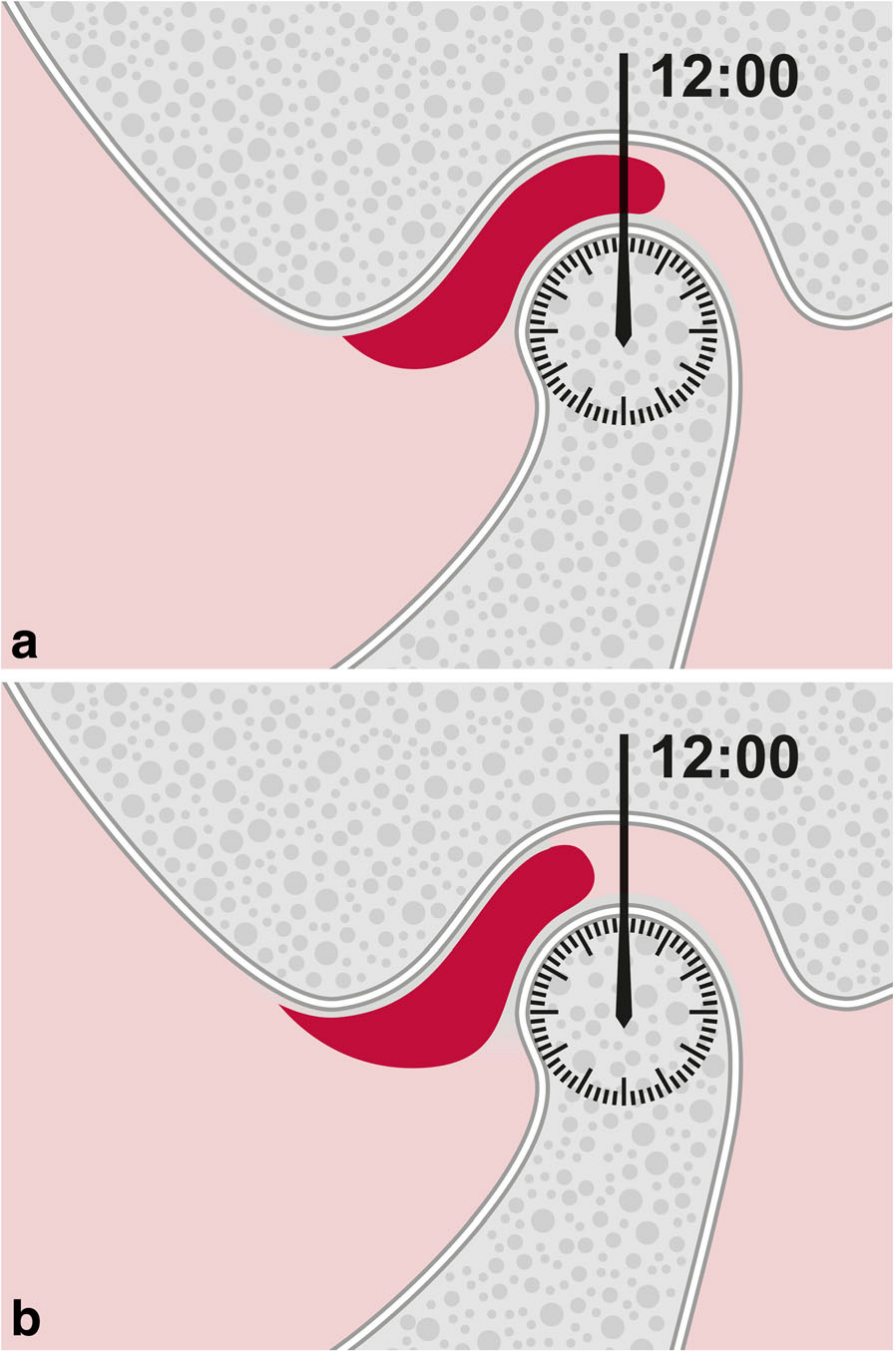
Evaluation of disc position at closed jaw, based on an imaginary clock located in the condylar head. **a** Textbook-like disc position: The posterior band is located at the 12 o’clock position (at the top of the mandibular condyle). **b** Anterior disc position: The posterior band is located anteriorly of the 12 o’clock position

Determination of the articular disc position at closed jaw was carried out following the criteria proposed by Orsini et al. [22]. A textbook-like “normal” disc position (e.g.,[23]), with the posterior band located at the top of the condyle (12 o’clock position) (Fig. 2a), was distinguished from an anterior location, where in intercuspal position (formerly: maximum intercuspation) the posterior band of the disc was located anterior to the 12 o’clock position (Fig. 2b).

#### Depth of the glenoid fossa at open jaw

**Fig. 3:**
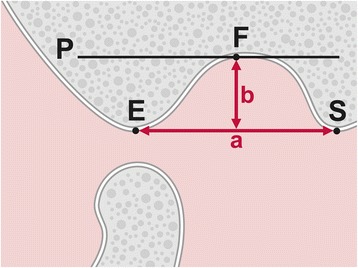
Points and lines used for measuring glenoid fossa depth. C: highest point of the condyle, E: most caudal part of the articular eminence, F: most superior aspect of the glenoid fossa, S: apex of the postglenoid spine, P: line parallel to the line SE touching F, a: distance from E to S = width of the fossa, b: perpendicular line from SE to P1 = depth of the fossa

The depth of the glenoid fossa was assessed during open jaw position by using the method introduced by Muto et al. [24]: A tangent was drawn from the most caudal part of the articular eminence (E) to the apex of the postglenoid spine (S). The distance between E and S, defined as “a”, corresponded to the width of the glenoid fossa. Subsequently, a parallel line to SE touching the most superior aspect (F) of the fossa was drawn (P). Finally, a perpendicular line was drawn from SE to P, which was equivalent to the depth (= height) of the fossa “b” (Fig. 3).

#### Statistical evaluation

The statistical analyses were carried out with SPSS version 21 (Statistical Package for the Social Sciences, Chicago, IL, USA). Frequency distributions were recorded for the condylar and disc positions at closed jaw. Student’s *t*-test [25] with independent (unpaired) samples was used as test of significance to detect differences between the age cohorts (1930 vs. 1950) and the sexes. The significance levels were set at 5%. First, the results from the measurement of the age cohorts were compared without differentiation of sexes, i.e., age cohort 1930–1932 versus age cohort 1950–1952. Subsequently, the age cohorts were compared by sex, i.e., men in cohort 1930–1932 versus men in cohort 1950–1952, and women in cohort 1930–1932 versus women in cohort 1950–1952.

#### Interrater reliability

To assess interrater reliability, the sagittal MRI views of the mandibular condyle and the articular disc of nine participants were evaluated at open and closed jaw positions independently by two authors (A.S. and M.S.) using the above-mentioned computerized digitizing software. The intra-class correlation coefficient [26] was used for the statistical computation. Based on the results of the reliability assessment (see Results: Interrater reliability), it was assumed that the measuring method was reliable. Therefore, the remaining MRIs were assessed by one rater only.

## Results

### Interrater reliability

Substantial interrater agreement could be reached (ICC: 0.7). Observational discrepancies were discussed until obtaining consensus.

### Position of the mandibular condyle at closed jaw

In both cohorts, condylar position was characterized by great variability (Tables 2 and 3). Altogether, 68 of 139 condyles (48.9%) were located centrically, whereas 71 (51.1%) were in an eccentric position. An absolute centric position was a rare exception and was present in less than 4% of individuals. In the female cohorts, a posterior position predominated (52.4%), while a centric position prevailed among men (56.6%). Nevertheless, neither age nor sex had a statistically significant influence on condylar position.

**Table 2:** Condylar positions (jaw closed) among women and men, respectively, determined according to the formula $\frac{\mathrm{posterior}\hbox{-} \mathrm{anterior}}{\mathrm{posterior}+\mathrm{anterior}}\times \kern0.5em 100\%\kern0.5em \left(n=139\right)$. There was no statistical significance in the relationship between the cohorts and the sexes, respectively

	Anterior	Absolutely centric	Centric	Posterior	Σ TMJs
Cohort 1930, women	2 (7%)	2 (7%)	9 (32%)	15 (54%)	28
Cohort 1950, women	3 (8%)	1 (3%)	13 (36%)	18 (50%)	35
Cohort 1930, men	10 (28%)	0	17 (47%)	8 (22%)	35
Cohort 1950, men	10 (24%)	2 (5%)	24 (57%)	5 (12%)	41
Σ TMJs	25 (18.0%)	5 (3.6%)	63 (45.3%)	46 (33.1%)	139

**Table 3:** Mean condylar position (jaw closed) among women and men, respectively, calculated according to the formula $\frac{\mathrm{posterior}\hbox{-} \mathrm{anterior}}{\mathrm{posterior}+\mathrm{anterior}}\times \kern0.5em 100\%\kern0.5em \left(n=139\right)$. There was no statistical significance in the relationship between the cohorts and the sexes, respectively

	Mean	SD	Min	Max
Cohort 1930, women	−20.7	23.5	−100	14.8
Cohort 1950, women	−11.4	19.1	−45.5	40
Cohort 1930, men	1.8	19.5	−40	40.3
Cohort 1950, men	5.1	13.6	−42.3	31.8

### Position of the articular disc at closed jaw

In three-forth of the TMJs, the most frequent position of the articular disc corresponded to the one described in textbooks. With other words, one out of four discs was “anteriorly displaced” (Table 4). The frequency of anteriorly located disc was twice higher in females than in males (22/63 vs. 13/76 = 34.5% vs. 17.1%; *p* < 0.05).

**Table 4:** Distribution of disc positions (jaw closed) among women and men, respectively (*n* = 139). There was no statistical significance in the relationship between the cohorts and the sexes, respectively

	Anterior	Textbook-like	Σ TMJs
Cohort 1930, women	7 (25%)	21 (75%)	28
Cohort 1950, women	15 (42%)	20 (56%)	35
Cohort 1930, men	9 (25%)	26 (72%)	35
Cohort 1950, men	4 (10%)	37 (88%)	41
Σ	35 (25.2%)	104 (74.8%)	139

### Depth of the glenoid fossa at open jaw

Comparison between the age groups revealed a statistically significant decrease (*p* < 0.05) of the depth of the glenoid fossa in both older cohorts: 6.9 mm (1950) vs. 6.1 mm (1930) among females, 7.8 mm (1950) vs. 6.3 mm (1930) among males (Table 5).

**Table 5:** Depth of the glenoid fossa (distance “b”) in mm (jaw open) among women and men, respectively (*n* = 130). Statistical significance was reached between the two female and the two male cohorts, respectively

	Mean	SD	Min	Max	*p*
Cohort 1930, women	6.1	1	3.6	7.7	<0.05
Cohort 1950, women	6.9	1.3	4.5	9.4	
Cohort 1930, men	6.3	2.5	3.0	16.5	<0.05
Cohort 1950, men	7.8	2	4.2	15.6	

## Discussion

### Methodology

Our population-based retrospective cohort study was based on imaging data stored in an electronic database. The records were from individuals of a representative population sample. We therefore assume that the participants were representative of that population, hence minimizing selection bias [27]. The typical disadvantages of patient-based retrospective studies, such as recall bias and information bias, did not affect the results because we made anatomical analyses on images, comparable to a histological examination. In our investigation, MRIs were used, which is the gold standard for imaging soft tissues [28]. In the temporomandibular joint area, MRIs are the images of choice to investigate disc position and optimize depiction of the condylar cartilage [29].

Conversely, for the investigation of hard tissues, such as condylar position and osseous changes, other imaging modalities, such as cone-beam computed tomography (CBCT), are more suitable [30, 31]. Yet, in this population-based study, CT scans would not have been allowed by the ethical committee to be used. Without doubt, this is a certain methodological limitation, although, on the other hand, MRIs have been shown to produce highly acceptable osseous changes in the TMJ.

As the slices had been selected identically in each subject, the comparability of the results can be assumed to be valid. Without doubt, the orientation of the slices has an influence on the measurement. Since the orientation was standardized, however, this approach seems to be acceptable. In this context, the shape of the condyle has certainly an impact on the measurement of the condylar position. While the calculated depth of the glenoid fossa based on MRI data might differ from the real anatomical depth, this possible bias is true for all measurements in all subjects and might be acceptable for this reason.

Using the technique FLASH imaging for rapid MRI [32, 33] ensures that acquisition times are reduced to seconds while spatial resolution is retained. For the purpose of our study, three-dimensional (3D) imaging [34] could not be realized due to unavailability of the existing 3D magnetic resonance scanner and the higher costs due to the longer acquisition time.

### Position of the mandibular condyle at closed jaw

We found a nearly 50% chance (71:68) of a deviation from a centric condylar position. These results corroborate findings of two systematic reviews on the position of the mandibular condyles of symptom-free dentate adults during intercuspal position [35, 36]. Stamm et al. [35], who analyzed the results from 49 imaging investigations covering the period until 2001, reported a range of anterior (cranioventral), (con)centric and posterior condylar positions. No statistically significant predominance of any specific condylar position was observed when the results of the six methodically best study articles were considered [35].

In a sequel of the Stamm et al. review, Türp and Walter [36] evaluated the four study reports [37–41] and two overview articles [42, 43] that were published between 2002 and 2012. The results of the four clinical studies attested to the wide interindividual variation. Centric positions were found less frequently than eccentric locations of the condyles. This conclusion stands in contrast to data from a CBCT study in Iran where among individuals without TMD history a centric condylar position was the rule [44].

As in an early investigation report by Pullinger et al. [45], more posterior condylar positions were observed among females and more anterior positions among men. Pullinger et al. [45] raised the question whether this finding could “partially contribute to the greater frequency of TMJ clicking in females”. Our study lends support to this hypothesis, because we identified twice as much anterior locations of the disc among women than among men.

Since in TMD patients an apparent association between condylar position and clinical findings worth to be treated is lacking [46], it appears that the often asked question of “the most physiological” or “the most optimum” condylar position in intercuspal position is more of academic interest than of clinical relevance. Instead, a relatively broad range of equally acceptable positions exists [36]. This assertion echoes the conclusion of Hugger et al. [37–41] who after analyzing 136 TMJs on MRIs stated: “Considering the great variability of the condylar position in patients with normal mandibular function and those with functional disorders of the stomatognathic system, the criterion “condyle-fossa relationship” seems more questionable than ever in the context of decision-making with regard to functional normality, adaptation or therapeutic requirements.” Hence, by defining “normality” as a “range of results beyond which target disorders become highly probable” [47] we concur with the statement by Pullinger that “non-concentric condyle positions can be compatible with normal function” [48].

### Position of the articular disc at closed jaw

Our observation that anterior disc locations are relatively frequent findings, is in concordance with imaging studies from the 1980s [49, 50] and 1990s [51]. More recent investigations have corroborated the assumption that so-called anterior disc displacements are common in normal populations. For example, in a community sample of 1643 individuals in Brazil, the prevalence of disc displacements as diagnosed clinically with the RDC/TMD was around 8% [52]. Not unexpectedly, higher values are found among patients. In a sample of 1603 adult TMD patients in Spain, the prevalence of the RDC/TMD diagnosis “disc displacement with reduction” was around 40% in each TMJ [53]. Among 520 consecutive TMD patients in Italy, RDC/TMD diagnoses of disc displacement were found in 42% of cases [54]. The values from the two European studies are close to those found in our investigation. TMJ sounds are widespread even among children and adolescents, as was recently reported in a systematic review which considered 17,051 individuals: a prevalence of 10% (95% confidence interval: 7.97–12.28) was calculated [55].

In the past, anterior disc displacements with reduction had been construed as the beginning of a cascade-like development leading to more severe TMJ pathology requiring therapy [56]. In the 1980s, Rasmussen [57] as well as Wilkes [58] proposed staging criteria for a progressive development of TMJ problems. While Rasmussen [57] differentiated six phases, Wilkes [58] distinguished five stages. Taking clinical, radiographic, and surgical findings into consideration, Wilkes postulated that a reciprocal TMJ clicking would eventually progress to pain of increasing frequency and intensity, leading to late stages of “chronicity” and “chronic restriction of [mandibular] motion” [58]. Yet, epidemiological population-based studies have challenged this view [59–61], which has also been corroborated by clinical findings among patient samples: In a recent cross-sectional multicenter study involving 614 cases with at least one TMD diagnosis, the association between TMJ intra-articular status (representing a transition from normal joint structure to TMJ disc displacement with or without reduction to degenerative joint disease) and patient-reported TMD impact (pain intensity, mandibular dysfunction, pain-related disability) was neither statistically significant nor clinically relevant [62]. In fact, the authors found no association between the intraarticular status of the TMJs and TMJ arthralgia or mandibular function [62]. Of course, these statements do not discard the possibility that a subset of individuals does indeed progress from clicking to osteoarthrosis; nonetheless, this is more an exception than the rule [48].

Accordingly, pain-free TMJ clicking (popping, snapping) during opening and/or closing [63], i.e. anterior disc displacement with reduction, may be interpreted as a “normal anatomical variant” presenting as subclinical symptom and/or sign that does not warrant further diagnosis or therapy [64] – as opposed to the alternative interpretation of an “abnormal joint pathology” [65]. The same applies to a pain-free disc displacement without reduction, which, in the absence of pain and/or mandibular dysfunction, should not be interpreted as a sign of pathology.

Although the line separating normal variation from pathological disorder is ill-defined, we agree with Ohrbach and Greene [64] that (a) pain associated with masticatory muscles or TMJs and/or (2) limitations of jaw movements (mostly restricted jaw opening), e.g. mandibular dysfunction, are those clinical symptoms and signs that require therapy, at least in most cases. Hence, *painful* TMJ clicking would warrant pain management, but irrespective of the presence of the clicking sound.

Imaging findings do not contribute to the clinical decision-making process because pain cannot be depicted on radiographs, whereas knowledge about the exact position of the articular disc appears to be unnecessary because there is no need for a therapeutic manipulation of the disc position. With other words: It is unnecessary to “confirm” a TMD-related diagnosis by sophisticated imaging. Instead, the principle of ALARA (“As Low As Reasonably Acceptable”) or ALADA (“As Low As Diagnostically Acceptable”) should be strictly observed [66].

### Depth of the glenoid fossa at open jaw

The age-dependent changes observed in our study may be interpreted as flattening of the temporal joint surfaces, particularly of the posterior slope of the articular eminence. Radiographic observations, such as flattening of the mandibular condyles or the articular eminence, are common among asymptomatic individuals [67, 68] as well as among patients with TMJ pain [69]. These morphological alterations may begin at early age: In a Chinese investigation, 711 of 4883 TMD patients (14.6%) aged between 11 and 30 years showed radiographic signs of TMJ osteoarthrosis [70]. The incidence of such osseous alterations continues with increasing age [71–73], probably as the consequence of long-term adverse loading of the intraarticular tissues [74], for example due to bruxism, while the role of loss of occlusal support on such morphological changes remains controversial [75].

Erosion with articular surface flattening is commonly interpreted as a sign of osteoarthrosis [76]. In the general population, the prevalence of TMJ osteoarthrosis lies between 2.4% [77] and 3.6% [78], whereas among patient populations with TMDs the estimated prevalence is 5.5% (based on seven studies representing 3,055 joints) [79]. In the joints of the human body, osseous changes, osteoarthritic pain, and impaired function generally increase with age [8, 80].

In contrast, the clinical symptoms of the typical TMD patient – a women between 18 und 50 year suffering from masticatory muscle pain – decrease remarkably during menopause and in older age groups [1]. Schmitter et al., for instance, found that among thirty 73 to 75 years old individuals drawn from a representative sample signs of TMJ osteoarthrosis were detected on gadolinium-enhanced MR images in 21 subjects (70%); yet, only one person (3.3%) reported TMJ arthralgia [9]. De Leeuw et al. [81] reported that 30 years after initial diagnosis of osteoarthrosis and internal derangement *clinical signs* were scarce and did hardly differ from those of asymptomatic controls, despite the fact that radiographic signs, such as flattening, were significantly more prevalent in former patients than in controls.

These findings support a statement of De Boever and Carlsson made more than two decades ago [82]. They noted that “many patients are unaware of the deviation in form and the case history is often negative for pain or acute dysfunction”, because “it may well be a remodeling due to changed function”, leading to pain-free adaptive changes of the osseous structures of the TMJ [83]. Therefore, TMJs with signs of osteoarthrosis are not necessarily associated with more symptoms of TMDs than TMJs without osteoarthrosis [84, 85]. On the other hand, clinical signs and symptoms of TMJ osteoarthritis are poorly correlated with osseous changes depicted with computed tomography [86] or CBCT [30]. Indeed, for a long time it has been known that on the basis of radiographic findings it is difficult to differentiate between osteoarthrosis and adaptive remodeling [87]. Thus, an irregular morphology of the TMJ, such loss of the rounded contour of the surface of the mandibular condyle and/or the posterior slope of the articular eminence, has been considered an indeterminate finding for DJD, which may represent a precursor to osteoarthrosis, but also variation of normalcy, a sign of aging, and/or adaptive remodeling [63, 88]. Indeed, since the classical papers by Sandstedt more than 120 years ago [89–91], it is part of the general knowledge of the dental community that continuous compression may lead to bone resorption, while traction (pull) may cause deposition of new bone (bone apposition). Therefore, we argue that repetitive mechanical load by compressive forces that exceeds the adaptive capacity of the TMJ may result in resorption of bone and flattening of formerly roundish joint surfaces of the mandibular condyle and the articular eminence [92, 93]. From a biological standpoint of plausibility, this would allow for a greater distribution of mechanical forces acting on the joint, for example during bruxing episodes, and could be interpreted as a bony adaptation [94].

## Conclusions

Based on the results of our investigation and following the arguments put forth in the discussion, we call for a re-interpretation of the significance and labeling of imaging findings and derived diagnoses because they may insinuate pathology which usually does not exist. Therefore, as far as the mandibular condyles are concerned, expressions such as “condylar displacement” or “eccentric position” for describing of a non-centric condylar position should be avoided. Besides, it may evoke negative cognitions among patients. This interjection also applies to the prevalent diagnoses “anterior disc displacement” and “degenerative joint disease”/“TMJ osteoarthrosis”. In most cases, these conditions are chance findings in the course of a clinical or imaging examination, with no disadvantageous outcomes for the individual. Two decades ago, relabeling of the expression “disc displacement” (in favor of “disc position”) had been already suggested [95]. We would like to extend our proposal by considering condylar position and TMJ morphology as well (Table 6). By doing so, we intend to protect non-patients against medicalization, overdiagnosis, and unnecessary therapy.

**Table 6:** Suggested new description of three traditional imaging and clinical findings

Traditional description	Suggested new description
Condylar displacement (eccentric condylar position)	(Anterior, posterior) condylar position
Anterior disc displacement	Anterior disc position
Osteoarthrosis	Adaptive remodeling (due to increased mechanical loading)

## Abbreviations

cm: Centimeter
CP: Condylar position
DC/TMD: Diagnostic Criteria for Temporomandibular Disorders
DJD: Degenerative joint disease
Hz/Px: Herz/Pixel [Bandwidth]
mm: Millimeter
MRI: Magnetic resonance imaging
ms: Millisecond
OPPERA: Orofacial Pain: Prospective Evaluation and Risk Assessment
RDC/TMD: Research Diagnostic Criteria for Temporomandibular Disorders
TMD: Temporomandibular disorders
TMJ: Temporomandibular joint
